# Feature Pyramid U-Net with Attention for Semantic Segmentation of Forward-Looking Sonar Images

**DOI:** 10.3390/s22218468

**Published:** 2022-11-03

**Authors:** Dongdong Zhao, Weihao Ge, Peng Chen, Yingtian Hu, Yuanjie Dang, Ronghua Liang, Xinxin Guo

**Affiliations:** 1 The College of Computer Science and Technology, Zhejiang University of Technology, Hangzhou 310023, China; 2 The College of Information Engineering, Zhejiang University of Technology, Hangzhou 310023, China; 3 The Institute of Deep-Sea Science and Engineering Chinese Academy of Sciences, Sanya 572000, China

**Keywords:** forward-looking sonar, sonar image segmentation, semantic segmentation, attention mechanism, convolution neural network

## Abstract

Forward-looking sonar is a technique widely used for underwater detection. However, most sonar images have underwater noise and low resolution due to their acoustic properties. In recent years, the semantic segmentation model U-Net has shown excellent segmentation performance, and it has great potential in forward-looking sonar image segmentation. However, forward-looking sonar images are affected by noise, which prevents the existing U-Net model from segmenting small objects effectively. Therefore, this study presents a forward-looking sonar semantic segmentation model called Feature Pyramid U-Net with Attention (FPUA). This model uses residual blocks to improve the training depth of the network. To improve the segmentation accuracy of the network for small objects, a feature pyramid module combined with an attention structure is introduced. This improves the model’s ability to learn deep semantic and shallow detail information. First, the proposed model is compared against other deep learning models and on two datasets, of which one was collected in a tank environment and the other was collected in a real marine environment. To further test the validity of the model, a real forward-looking sonar system was devised and employed in the lake trials. The results show that the proposed model performs better than the other models for small-object and few-sample classes and that it is competitive in semantic segmentation of forward-looking sonar images.

## 1. Introduction

As humans continue to explore the oceans, they are constantly conducting research into marine resources. Underwater imaging technology is an important aspect of this work [1]. However, it is difficult for optical imaging devices to achieve good results owing to the turbidity of seawater and low light levels [2,3,4]. In contrast, sonar sensors are well suited to these conditions [5,6,7]. For example, multibeam forward-looking sonar provides underwater images by using new two-dimensional sonar imaging technology to record high-speed motion [8]. This approach has the advantage of using equipment that is portable and easy to operate, making it ideal for underwater observations. Thus, the demand for forward-looking sonar equipment has been increasing in recent years. However, sonar also has some limitations. For example, images can be affected by noise from the imaging mechanism and complex environments [9,10,11]. This interference can result in blurred target areas and complex edge information, which seriously affect subsequent image processing [12]. Despite the existence of synthetic aperture sonar, which can provide high-resolution sonar images and is nearly independent of frequency and target range [13,14], it still has speckle noise in its images [15], which does not facilitate us to develop further studies of the images.

As sonar imaging applications have developed, there has been widespread research into sonar image segmentation technology [16,17]. The main purpose of sonar image segmentation is to divide images into several specific regions with unique properties and to identify targets of interest [18]. Moreover, sonar image semantic segmentation is required to identify different targets for segmentation [19]. Thus, it can help researchers to identify the important parts of images quickly, and it has important practical applications. At present, sonar image segmentation methods can be roughly divided into five categories based on: (1) thresholding, (2) edge detection, (3) Markov random field models, (4) clustering algorithms, and (5) artificial neural networks [20]. Liu et al. [21] proposed a threshold segmentation method for underwater linear target detection based on prior knowledge, and they achieved good segmentation quality and computation time by analyzing the threshold variation. Wu et al. [22] introduced a fractal coding algorithm for regional segmentation of sonar images, which improved the segmentation speed. Villar et al. [23] proposed a side-scan sonar target segmentation method, which introduced the order statistic-constant false alarm rate (OS-CFAR) and sliding windows to achieve segmentation. This method is less sensitive to scattered noise than other methods, and it can achieve better segmentation accuracy. The thresholding segmentation method is simple and easy to implement, but accurate results can be obtained only when there are significant variations in grayscale images. Karine et al. [24] extracted the features of textured images using generalized Gaussian distribution and α-stable distribution. They showed that their method is applicable to sonar image segmentation, but its use in high-noise scenarios is limited because it is a frequency domain operation. Kohntopp et al. [25] segmented specific objects in sonar images using an active contour algorithm, and their method can adapt to the intensity distribution characteristics of sonar images. Li et al. [26] proposed a new active contour model for image segmentation. This approach embeds a local texture neighborhood region and defines its structure with respect to the noise and object boundary pollution in the image. They also introduced a Bayesian framework that embeds a Markov random field model and local texture information to manage intensity inhomogeneities. Song et al. [27] proposed a side-scan sonar segmentation algorithm based on a Markov random field and an extreme learning machine, and their method showed good segmentation results for sonar data. However, although Markov fields use local information effectively, the use of global information is insufficient. Abu et al. [28] proposed a sonar image segmentation method that combines the level set and lattice Boltzmann methods. They achieved more accurate segmentation by dividing the segmentation task into two subtasks. Xu et al. [29] proposed an enhanced fuzzy segmentation algorithm based on a kernel metric and improved the segmentation accuracy by introducing local spatial and statistical information. This method is suitable for sonar images with inhomogeneous intensity and complex seafloor textures.

These methods have disadvantages of high algorithm complexity, slow recognition speed, and high image quality requirements [15,30,31,32], so there is an urgent need for more efficient sonar image segmentation methods. Neural-network-based image segmentation has become a popular research direction [33] as it has excellent performance in complex image segmentation. This is discussed in detail in Section 2.

Forward-looking sonar devices provide real-time sonar images for underwater target detection, navigation, surveillance, and inspection [34,35,36]. In combination with semantic segmentation algorithms, forward-looking sonar can present the underwater scene clearly, providing an important basis for target localization and identification [37]. At present, semantic segmentation of forward-looking sonar images has the following challenges [38]: (1) serious noise interference, which makes it difficult to segment target areas accurately, especially when they are small; and (2) many images are required to obtain sufficient data to achieve high segmentation accuracy, and improvements are required to achieve high accuracy from a limited number of images. Recently, deep-learning-based semantic segmentation has demonstrated excellent performance. U-Net was obtained by extending and modifying a full convolutional network [39]; it consists of two parts: a contraction path to obtain contextual information and a symmetric expansion path for accurate locating. This approach requires a small amount of training data and good results can be achieved quickly, so it is often used in medical image segmentation and has attracted research interest [40]. There are some similarities between sonar and medical images as they both obtain information about a target using ultrasound [41]. Therefore, this work was based on U-Net.

This study integrates the residual model [42] into U-Net in order to improve the network and address the difficulties associated with deep model optimization. To enhance the integration of semantic messages (decode part) and shape messages for objects of different sizes, we introduce a multi-layer feature fusion algorithm that combines U-Net and multi-layer features to reduce the possibility of mis-segmentation. Moreover, to better integrate deep semantic and shallow contour features, and to improve the recognition of important features, an attention method that allows the model to consider both semantic features and shallow contours is demonstrated. Thus, a feature fusion network based on U-Net called Feature Pyramid U-Net with Attention (FPUA) is presented. FPUA solves the difficulties with optimizing depth models by introducing the residual module and introduces the feature pyramid network module and attention structure to improve the accuracy of semantic segmentation for small object classes. In summary, the proposed model can extract semantic information from forward-looking sonar images better than existing models.

The main contributions of this work are as follows. (1) A network model specifically for semantic segmentation of forward-looking sonar images, called FPUA, is proposed. It uses a fused feature pyramid method to improve the overall segmentation accuracy by synthesizing deep semantic and shallow detail information. (2) A fused attention structure is proposed to provide different weights to different features in the feature pyramid, which helps to improve segmentation accuracy for small targets. (3) Using the marine-debris-fls-datasets dataset, the proposed model is compared to mainstream models. This shows that the proposed model can achieve good segmentation results overall and for small objects. Moreover, it indirectly promotes recognition of small samples because the classes with few samples mainly include small objects. (4) We have produced datasets based on real environmental data and have demonstrated that our models can achieve good results in real environments by comparing them with mainstream models.

## 2. Related Work

The main aim of this study is to achieve semantic segmentation for forward-looking sonar images. The proposed network will improve the ability to capture semantic information from noisy images and show better segmentation performance for small objects. This section will describe existing research applying deep learning to sonar images, then discuss common semantic segmentation methods.

### 2.1. Current State of Sonar Imaging Research

Many scholars have investigated application of deep-learning-based methods for sonar image analysis. These methods use sonar images as training data to identify intrinsic laws and representation levels, which avoids the need for researchers to conduct in-depth analysis of image features and reduces accuracy loss caused by improper feature selections [30].

Fan et al. [43] proposed a deep-learning-based method of sonar image target detection that used a series of residual blocks to construct a 32-layer feature extraction network. This network structure improved detection of sonar image targets and reduced the number of training parameters required. However, compared with other methods, it did not improve the quality of detection. Song et al. [44] proposed a convolutional neural network for side-scan sonar that could utilize both local and global features through cropping layers and that did not require additional convolutional parameters. Their method is mainly used for target detection in side-scan sonar, and it can satisfy real-time target detection tasks. Wang et al. [45] proposed a real-time semantic segmentation network for side-scan sonar images. They improved the performance using deep separable convolution and 2-way branching and implemented a corresponding decoder network to recover details of the target. This method can satisfy certain real-time requirements with high segmentation accuracy, and the experimental results show that it can also satisfy real-time requirements for side-scan sonar images.

### 2.2. Semantic Segmentation Based on Deep Learning

Semantic segmentation is a fundamental task in computer vision in which image information is separated into different semantically interpretable classes [46]. Although target detection methods can help to determine the edges of identified entities, semantic segmentation can label objects at pixel level, which provides a more detailed understanding of the image than image classification or target detection. Nowadays, mainstream semantic models include U-Net, DeepLabV3, and PSPNet.

The FPN model is a classical target recognition model that was proposed by Lin et al. in 2017 [47]. It effectively solves the problem of predicting different-size targets. Moreover, the FPN model improves utilization of image information by combining shallow features and deep semantic information, and this method is also applicable to semantic segmentation [48,49]. The U-Net model was proposed by Ronneberger et al. [39]. It is similar to the FPN model in that it performs feature fusion to obtain richer semantic information. However, because U-Net is applied directly to semantic segmentation, it needs to judge the class information of each pixel, so multiple feature fusions are required to make the features richer and the semantic segmentation more accurate. The U-Net model can achieve good segmentation with fewer data samples than previous models. Based on U-Net, Zhou et al. [50] proposed the U-Net++ model. They argued that it is inappropriate to use a skip connection to combine the shallow features of the encoder directly with the deep features of the decoder, as in U-Net, because this generates a semantic gap. Instead, they connected the skip path through a series of nested, dense skip paths, with the aim of reducing the semantic gap between the feature maps of encoder and decoder sub-networks. Subsequently, Huang et al. [51] proposed the U-Net3+ model based on U-Net++, in which each decoder layer incorporates small- and same-scale feature maps from the encoder, and large-scale feature maps from the decoder, to capture both fine- and coarse-grained semantics at full scale.

The PSPNet model was proposed by Zhao et al. [52]. The main innovation of this model lies in the proposed spatial pyramid pooling module. In segmentation tasks, the size of the perceptual field is indicative of the ability to use contextual information, and the empirical perceptual field of a neural network is much smaller than the theoretical one, especially for deep scene networks [53]. Thus, the model provides an effective global contextual prior through a hierarchical global prior containing contextual information for different scales and different sub-regions. The DeepLab series is a series of semantic segmentation algorithms proposed by the Google team, and DeepLab v3+ is based on DeepLab v3, which was proposed by Chen et al. [54]. It borrows the encoder–decoder architecture from networks such as the FPN, implements feature map fusion across blocks, and uses group convolution to improve the operation speed. Thus, the DeepLab v3+ model contains more shallow information and optimizes the segmentation effect for segmented edges.

The above methods can segment images well in simple marine environments, but their performance is poor when applied to complex images with high noise and small object areas. This is mainly because shallow detail information and deep sematic information from the graph cannot be fully utilized. To address this, we propose a model that incorporates more semantic information.

## 3. Proposed Approach

**Figure 1:**
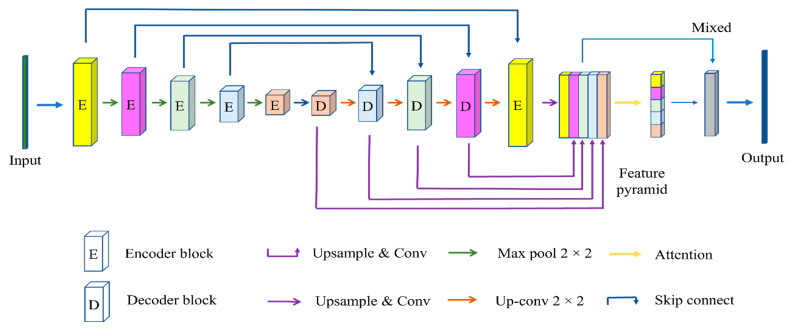
Diagram showing the overall structure of FPUA, where different colors indicate different sizes of feature information.

This section will introduce the proposed model, FPUA. The overall framework is shown in Figure 1. FPUA includes the U-Net module, residual block, feature pyramid network module, and attention structure. The U-Net module includes the encoder, decoder, and skip connection modules. U-Net has proven to be an efficient semantic segmentation architecture. It uses skip connections in the reconstruction phase to pass feature maps from the same-level encoder, which makes it very convenient for segmentation tasks that require precise localization. Therefore, U-Net was chosen for the backbone of the proposed model, and the residual block is introduced to supplement the feature information lost during the convolution process. To improve the segmentation accuracy of the network model, a feature pyramid network is used to fuse the features of each decoder so that more semantic information can be obtained. Different decoders have different effects on the results. To improve the segmentation accuracy of the model for small objects, an adaptive attention scheme is proposed that dynamically assigns different decoder weights; this improves utilization of semantic information.

### 3.1. Residual U-Net

**Figure 2:**
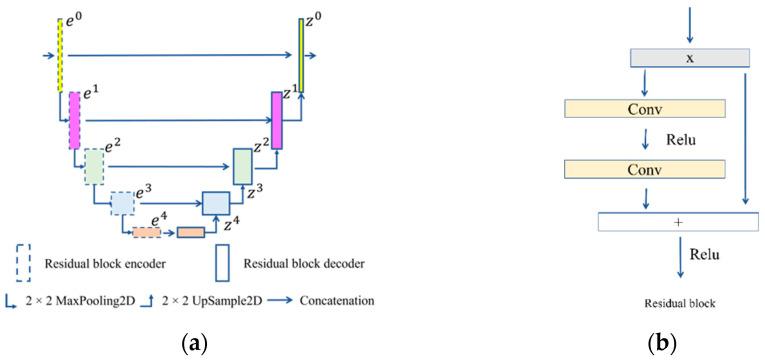
Residual U-Net structure. (**a**) U-Net structure and (**b**) residual block.

The main structure of U-Net is composed of two parts, as shown in Figure 2a, with an encoder on the left and a decoder on the right. The encoder consists of five submodules, which each contain a downsampling layer implemented by a max pool. The resolution of the input image is 320 × 480, and the resolutions of modules 1–5 are 320 × 480, 160 × 240, 80 × 120, 40 × 60, and 20 × 30, respectively. The decoder consists of five submodules, and the resolution is increased sequentially through upsampling until it matches the resolution of the input image. The encoder process can be expressed as

(1) $$e^{i}=E^{i}(e^{i-1})$$

where *e^i^* denotes the result of encoder at layer *i*, E*^i^* denotes the encoder structure of U-Net at layer *i*, and the decoder process can be expressed as

(2) $$z^{i}=D^{i}(e^{i},z^{i+1})$$

where *e^i^* denotes the result of encoder at layer *i*, E*^i^* denotes the encoder structure of U-Net at layer *i*.

The residual block uses the residual connections to fuse the convolved results with the original input features to improve the performance and optimization efficiency of the network. Its structure is shown in Figure 2b. The residual block in U-Net splices the outputs from submodules with the same resolution, which preserves semantic information at a certain scale.

### 3.2. Feature Pyramid Network

**Figure 3:**
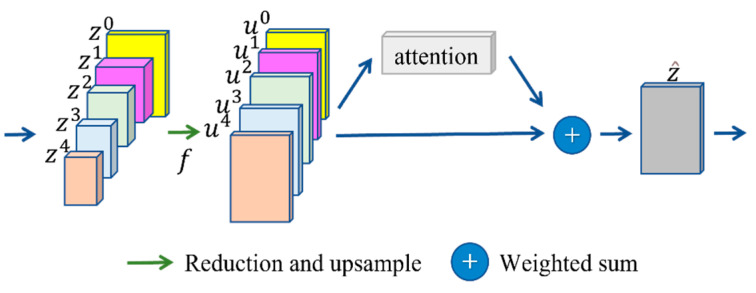
Feature pyramid network structure.

The shallow network focuses more on shape information, and the deep network focuses more on semantic information. Thus, the shallow network can help segment the region of an object accurately, and the deep network can help segment the target class accurately. The feature pyramid can include pooling, and different pooling can generate new feature maps with different semantic sizes. However, pooling lacks semantic information. In contrast, U-Net uses skip connect and deep decoder information to obtain a feature map, so some detail information is lost. Therefore, a feature pyramid incorporating multi-level semantic information, which will improve the accuracy of sample segmentation, is proposed. The proposed structure is shown in Figure 3.

Different decoder feature maps have different sizes and shapes, so dimensionality reduction and upsampling are used to align the different feature maps with the last feature.

**Figure 4:**
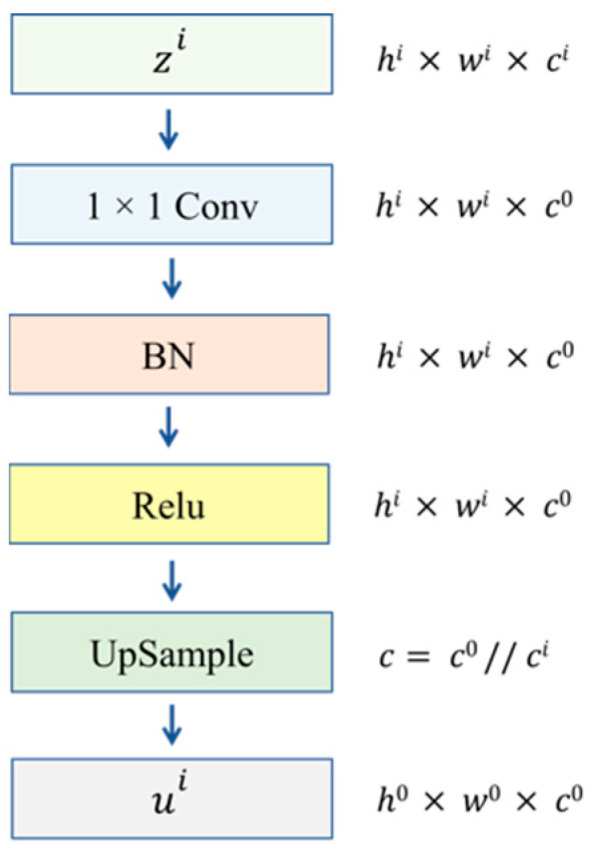
Reduction and sampling.

To reduce the number of parameters and memory usage, 1 × 1 conv is used as the dimensionality reduction method, where the dimensionality reduction operation is first applied to the input, followed by upsampling. This process is shown in Figure 4. First, *z^0^* is taken as the final target, then *z^i^* is downsampled from *c^i^* to *c*^0^ and upsampled by a factor of *c*^0^//*c^i^* to obtain *u^i^*. This process can be expressed as

(3) $$u^{i}=\mathrm{Upsample}({\mathrm{Conv}}_{1\times 1}(z^{i}))$$

where *z^i^* denotes the result of U-Net layer *I* decoder, *u^i^* is the result after dimensionality reduction and upsampling.

To prevent information loss, features are traditionally fused by concatenating them together. Although concatenation retains the information of each feature, it generates intermediate variables that occupy a great deal of explicit memory. In addition, different features are not assigned different weights, and they are considered to contribute equally to the result.

### 3.3. Attention Structure

An attention-based feature fusion method is proposed as a means of making the model pay attention to different feature information. First, the weights of different features are obtained through the attention structure. This process can be expressed as

(4) $$A=\mathrm{Att}(C)\;c^{i}\in C$$

where *C* is the result after pooling, *A* denotes a vector of batch size × 5 dimensions, and each value is greater than 0 and the sum is 1. Then, different features are fused into a new feature, which can be expressed as

(5) $$\hat{z}=\sum a^{i}\times u^{i}\;a^{i}\in A,$$

where *a^i^* is the attention weight corresponding to different scales, *u^i^* is the characteristic at different scales, and $\hat{z}$ is the overall feature information after fusing multi-scale attention.

The features incorporating multiple layers of semantic information are then used to predict the segmentation results.

**Figure 5:**
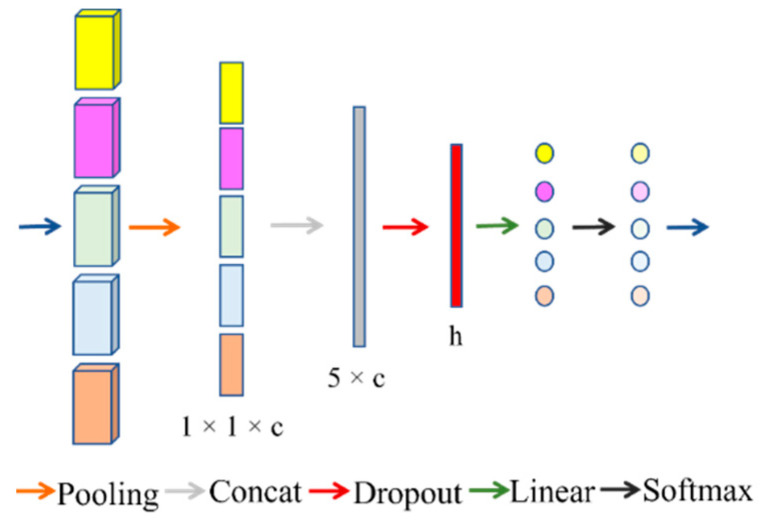
Attention structure. The color of the input block corresponds to the different dimensions of Figure 1; after linearization, the different colors represent different attention scores.

The attention module is proposed as a way to fuse multiple layers of feature information, as shown in Figure 5. Inspiration is taken from SENet [55], and a new attention structure is proposed. The purpose of this structure is to augment important features and attenuate unimportant ones so that the extracted features are more directed. First, pooling is used to extract channel information through feature compression. Then, the compression information for five features is connected to 5 × c two-dimensional information. Finally, the attention weights of each feature module are predicted using a multilayer perceptron (MLP) network. The MLP module contains a hidden layer and a dropout layer, which compresses the channel information for each feature, fuses them, and obtains attention through the fully connected layer.

## 4. Experiment and Analysis

In this section, we will first present the analysis of the model using the water tank dataset, and then we build the dataset for the real environment. With this dataset, the segmentation effect of our model in the real environment will be verified. Finally, we use our own developed forward-looking sonar equipment for image acquisition and processing to further demonstrate the feasibility of our method through a real-world environment with high noise. Through these three experiments, the performance of the FPUA model in different noisy environments will be demonstrated. The information of the three datasets is shown in Table 1.

In the experimental part, the Adam optimizer [56] is used, and the parameters of all network models are kept consistent, where learning rate = 0.002, decay = 0, 1st exponential decay rate is 0.9, and 2nd exponential decay rate is 0.99. The epoch of each network in all experiments is 100 generations, and the model with the highest score in the validation set is taken as the optimal model for the current network, and the model is used in the test set to obtain the actual segmentation score. Table 2 shows the protocols and parameters of the baseline methods.

### 4.1. Tank Dataset

**Figure 6:**
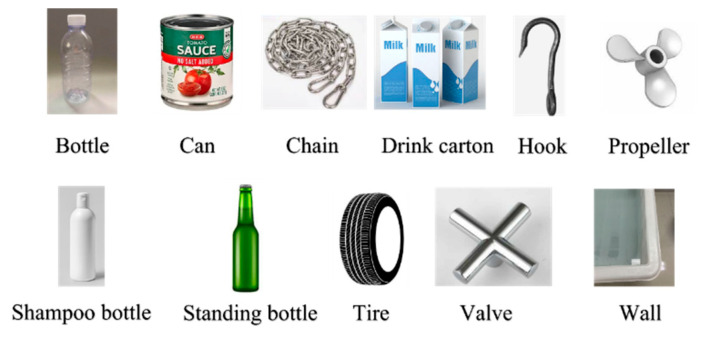
Examples of objects belonging to various classes.

The data used in this study consisted of 1868 fls images acquired by the ARIS Explorer 3000 sensor presented by Alejandro et al. [38]. The data were collected in a (W, H, D) = (3 × 2 × 4) tank with a sonar frequency of 3.0 MHz. The sonar has 128 beams with a field of view of 30° × 15° and a spacing of 0.25° between beams. The sonar spatial resolution is 2.3 mm per pixel in close range and almost 10 cm per pixel at the far range. The sonar was installed above the water tank and had a pitch angle between 15° and 30°. They were all grayscale images 480 × 320 pixels in size, and the class information was obtained by categorizing each pixel by class. All the targets were divided into twelve classes: bottle, can, chain, drink carton, hook, propeller, shampoo bottle, standing bottle, tire, valve, wall, and background, as shown in Figure 6. The bottle class included horizontally placed glass and plastic bottles; the can class included a variety of metal cans; the chain class was a one-meter-long chain; the drink carton class consisted of juice and milk boxes placed horizontally; the hook class included small metallic hooks; the propeller class was a metal propeller, like those used in small boats; the shampoo bottle class was a shampoo bottle placed vertically; the standing bottle class consisted of a standing glass beer bottle; the tire class was a small rubber tire placed horizontally; the valve class consisted of a metal valve; and the wall class included boundary locations. Not all the images in the dataset were clearly visible, and some were unclear owing to noise.

**Figure 7:**
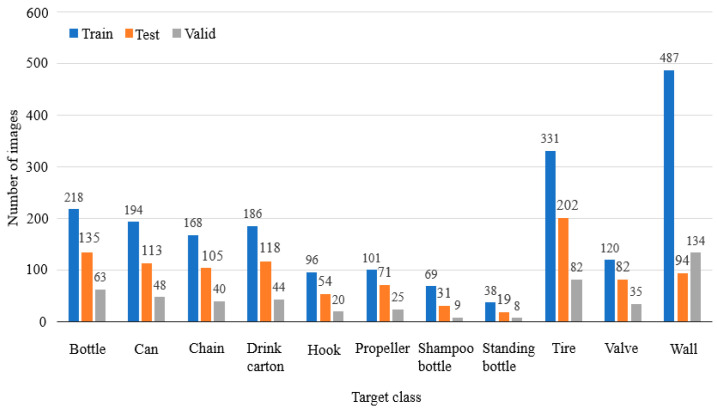
The number of images of each class in the training set, test set, and validation set.

The data were divided randomly to provide 1000 images in the train set, 251 images in the validation set, and 617 images in the test set. The random division ensured that the data were evenly distributed across each set, and the number of images from each class in each set is shown in Figure 7.

Analysis of the randomly divided data revealed that the dataset suffered from sample imbalance, which is consistent with the existence of majority and minority classes of targets in the marine environment. The proportions of each class are shown in Figure 7. Among the classification data, the hook, propeller, shampoo bottle, and standing bottle classes accounted for the smallest proportions of the samples.

**Figure 8:**
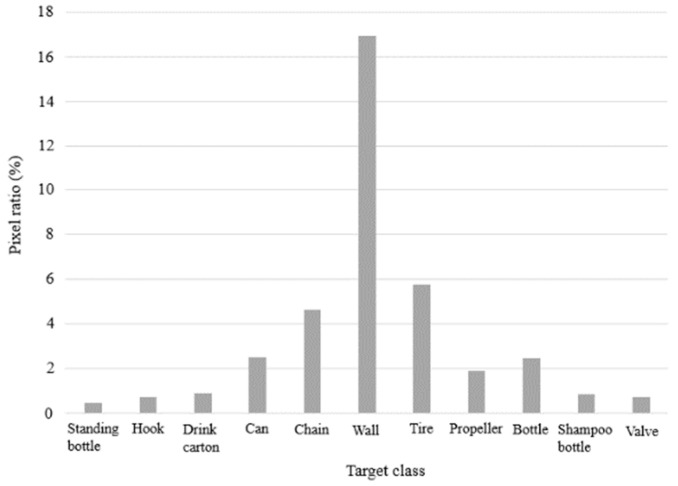
Pixel ratio for each target class.

To judge the proportion of image pixels belonging to targets of each class, the pixel distribution was obtained for each class, as shown in Figure 8. This shows that all the classes, except the wall class, occupied a relatively small number of pixels, among which the drink carton, hook, shampoo bottle, standing bottle, and valve classes occupied the smallest proportions, so these are small-target classes. The data show that most of the few-sample classes contain small objects, so we assumed that the proposed model would also improve the segmentation of few-sample classes. The subsequent analysis will consider the effect of few samples and small objects on the experimental results.

All the experiments in this study used dice loss as the loss function, which is valid for sample imbalance. To analyze the segmentation effect of the proposed model on the dataset, we used the mean intersection over union (mIoU). The IoU is commonly used to evaluate semantic segmentation, and it is an important reference metric. The mIoU is used to obtain the segmentation accuracy of pixels in each class by calculating the IoU and then merging to obtain the overall segmentation accuracy afterwards. Considering that the background occupies a relatively large area and does not contain specific semantic information, it should not appear as an independent class. Therefore, this study only counted information from the eleven remaining classes and analyzed them using the mIoU.

### 4.2. Tank Experimental Result

**Figure 9:**
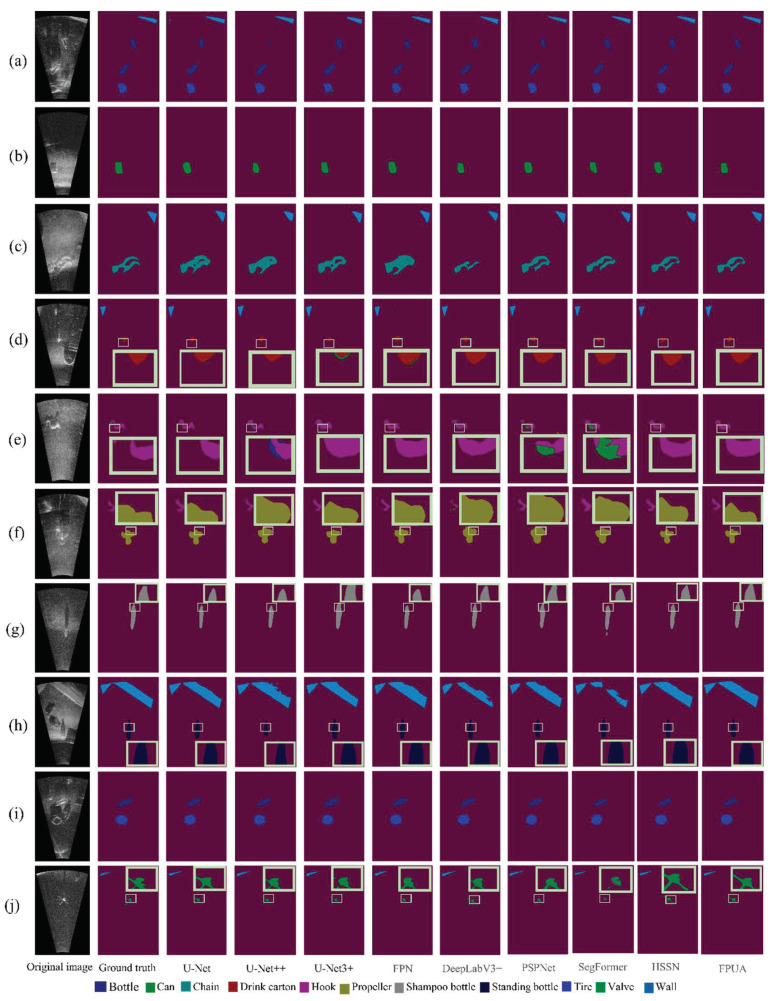
Segmentation results by target class for different models. Subfigure (**a**) shows the segmentation effect of bottle and wall, subfigure (**b**) shows the segmentation effect of can, subfigure (**c**) shows the segmentation effect of chain, subfigure (**d**) shows the segmentation effect of drink carton, subfigure (**e**) shows the segmentation effect of hook, subfigure (**f**) shows the segmentation effect of propeller, subfigure (**g**) shows the segmentation effect of shampoo bottle, subfigure (**h**) shows the segmentation effect of standing bottle, subfigure (**i**) shows the segmentation effect of tire, subfigure (**j**) shows the segmentation effect of wall. Subfigure (**h**) shows the segmentation of standing bottle, subfigure (**i**) shows the segmentation of tire, and subfigure (**j**) shows the segmentation of valve. The yellow box in the figure represents the enlargement of some details.

Figure 9 shows the segmentation results for the dataset with different models. To represent the segmentation effect clearly, some samples and small objects are labeled. The proposed model was compared with the U-Net, U-Net ++, U-Net 3+, FPUA, FPN, DeepLabV3+, and PSPNet models. The results show that the proposed model provided more detailed segmentation than the other models when there were few samples (see Figure 9e–h). It also showed better performance in contour segmentation for small objects (see Figure 9d,e,g,h,j). Thus, the proposed model can improve the accuracy of semantic segmentation for few-sample and small-object classes. It also showed good segmentation performance for other classes. Therefore, the proposed model can be used to improve the accuracy of semantic segmentation for forward-looking sonar images.

The effect of semantic segmentation was analyzed in terms of the metrics. Table 3 shows that the proposed model has significantly better accuracy than the other models for the chain, hook, shampoo bottle, and valve classes, and similar accuracy to the optimal models for the other classes. We also find that the transformer-based SegFormer model does not achieve good results due to the amount of data [59] and noise.

Consider the few-sample classes, that is, the hook, propeller, shampoo bottle, and standing bottle classes. For the hook class, the segmentation accuracy of the proposed model is similar to the best model. For the propeller class, the proposed model had similar segmentation accuracy to the U-Net3+ model but still improved the accuracy by at least 1% compared to the other models. For the shampoo bottle class, the proposed model improved the segmentation accuracy by 1.2%. For the standing bottle class, the proposed model improved the segmentation accuracy by approximately 3% compared to the other models, except for U-Net ++. The average mIoU for these classes was used as the reference metric for the few-sample classes, and the proposed model improved the average segmentation accuracy by 1.5%. This indicates that it can achieve relatively high-accuracy segmentation when trained with few samples. The main contribution comes from the fact that there were few samples of small objects, which proves that the proposed model has good segmentation performance for small objects with few samples.

Next, consider the small-object classes. Table 3 shows that the proposed model can achieve great segmentation results for small objects. The small-object classes included the drink carton, hook, shampoo bottle, standing bottle, and valve classes. The results for the hook, shampoo bottle, and standing bottle classes were discussed above. For the drink carton class, the proposed model achieved a segmentation result similar to that of the U-Net3+ model and had segmentation accuracy approximately 3% better than the other models. For the valve class, the proposed model has similar accuracy to HSSN and improved the segmentation accuracy by approximately 2%. The small-object mIoU was obtained by taking the average for all the small-object classes, and the proposed model improved the accuracy by approximately 1%. This demonstrates that the proposed model had excellent performance for small-object classes.

In summary, the proposed model achieved good semantic segmentation of forward-looking sonar images for the few-sample and small-object classes and also achieved high segmentation accuracy for the other classes.

### 4.3. Ablation Experiment

To demonstrate that our modules affect the results, an ablation experiment was conducted using the proposed model. The ablation experiment considered the effects of the residual block, FPN module, and attention structure on the model performance. The experiment included six models. First, the residual module was reserved and the effects of the other two modules on the experimental results were investigated. Then, the residual block was removed, and the effects of the FPN and attention modules on the experimental results were investigated. The names of these models are shown in Table 4.

**Figure 10:**
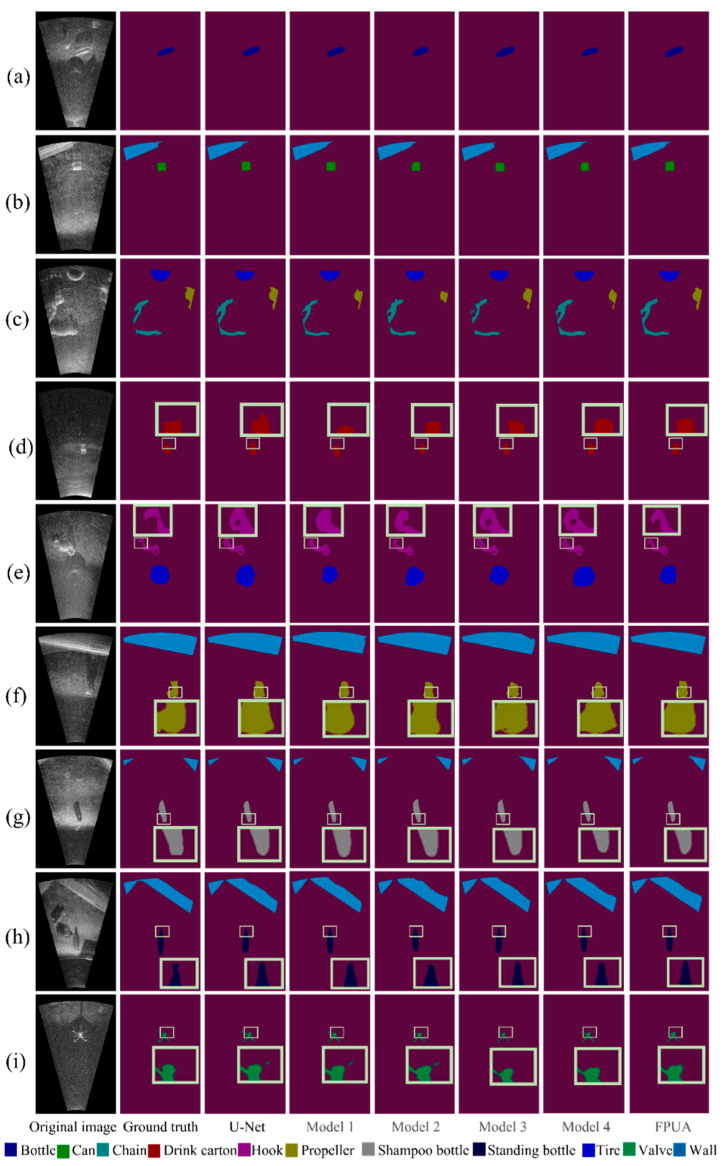
Segmentation results for each model in the ablation experiment. Subfigure (**a**) shows the segmentation effect of bottle, subfigure (**b**) shows the segmentation effect of can and wall, subfigure (**c**) shows the segmentation effect of chain, subfigure (**d**) shows the segmentation effect of drink carton, subfigure (**e**) shows the segmentation effect of hook and tire, subfigure (**f**) shows the segmentation effect of propeller, subfigure (**g**) shows the segmentation effect of shampoo and partition effect of shampoo bottle, subfigure (**h**) shows the partition effect of standing bottle, and subfigure (**i**) shows the partition effect of tire. The yellow box in the figure represents the enlargement of the details.

Figure 10 compares the segmentation results of each model in our experiments. To compare the effect of each module, the few-sample and small-object classes are labeled in the figure. Comparing each model in the figure shows that the residual module, feature pyramid module, and attention structure improve the segmentation accuracy, and the results are closer to the original image.

The effect of reserving the residual module is shown in Table 5. Comparing U-Net and model 1 shows that the residual block produced a greater improvement in the overall segmentation effect. The U-Net network slightly outperformed model 1 in the can, drink carton, and shampoo bottle classes, whereas it performed worse in the other classes, up to 13.6% in the standing bottle class, which shows that the residual block can improve the overall performance of the network.

The effect of the FPN module on the segmentation accuracy was also investigated. Comparing model 1 and model 2 shows that the overall segmentation accuracy was improved slightly. The segmentation effect was poor for the chain and tire classes, but the performance was better for the other classes, and the accuracy in the propeller category was improved by approximately 6%. Therefore, introduction of the FPN module is beneficial to the network and improves the overall segmentation effect. Moreover, a good leaning effect and high segmentation accuracy can still be achieved when there are few samples.

Finally, the effect of the FPN module combined with the attention structure on the segmentation accuracy was investigated. Model 2 was compared with the proposed network model FPUA. Model 2 had better results in two classes, the bottle and propeller classes, but the difference in segmentation accuracy was less than 1%. This is in line with the expectation that the attention structure would improve the segmentation accuracy of the network for small objects, so introduction of the attention structure is beneficial to the overall segmentation accuracy of the network.

### 4.4. Marine Dataset

The above experiments demonstrate the good performance of our model. To validate the performance of the model in the marine environment, we have used data from an open-source website for dataset production (http://www.soundmetrics.com/, accessed on 1 August 2022). The site performed data acquisition using ARIS Explorer 3000, which contains sonar video data in tilt and roll modes. For the video resource, we acquired only one frame per second and labeled the data with LabelMe, and a total of 3116 images were labeled. The data can be divided into 12 classes, and the specific class divisions are shown in Table 6.

**Table 6:** Selection of semantic classes available in our dataset.

Class ID	Object	Description
1	Schools of fish	A school of small fish. (Figure 11a)
2	Nurse shark	A fish with a large pixel ratio. (Figure 11b)
3	Divers	Underwater swimmers. (Figure 11c)
4	Pipe leakage	Gases leaking from pipelines. (Figure 11d)
5	Ammunition box	Rectangular shaped ammunition box. (Figure 11e)
6	Tire	Round tire. (Figure 11f)
7	Mesh box	Boxes with mesh holes. (Figure 11g)
8	Spinning umbrella	A round umbrella. (Figure 11h)
9	Salmon	A species of fish, more elongated. (Figure 11i)
10	Barrel	Horizontally positioned barrel. (Figure 11j)
11	Propeller	Spinning propellers. (Figure 11k)
12	Sunken aircraft	Underwater aircraft wreckage. (Figure 11l)

**Figure 12:**
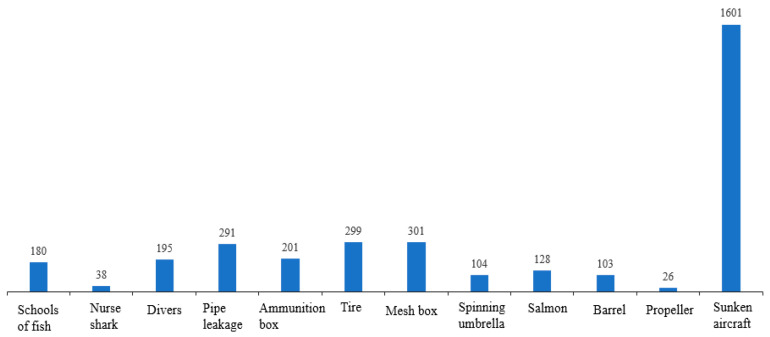
Number of images per segmentation class.

The number of each class is shown in Figure 12. In our experiments, we do not introduce the background as a class, and, from the figure, we can see that sunken aircraft makes up the largest proportion of the dataset, while the number of images of nurse shark and propeller is relatively small.

**Figure 13:**
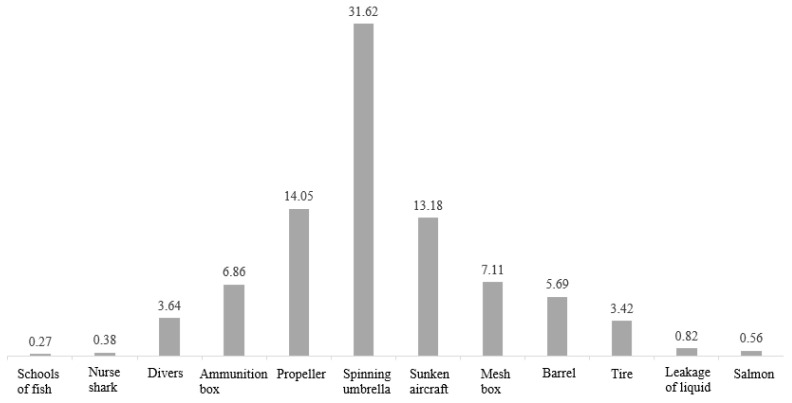
Distribution of pixels per class in real ocean data.

Since most of the classes have a small number, here, we mainly distinguish the small object classes and do not consider the few sample classes separately. The specific pixel distribution is shown in Figure 13. Among them, schools of fish, nurse shark, pipe leakage, and salmon have fewer pixels and belong to the small objects class.

### 4.5. Marine Experimental Results

**Figure 14:**
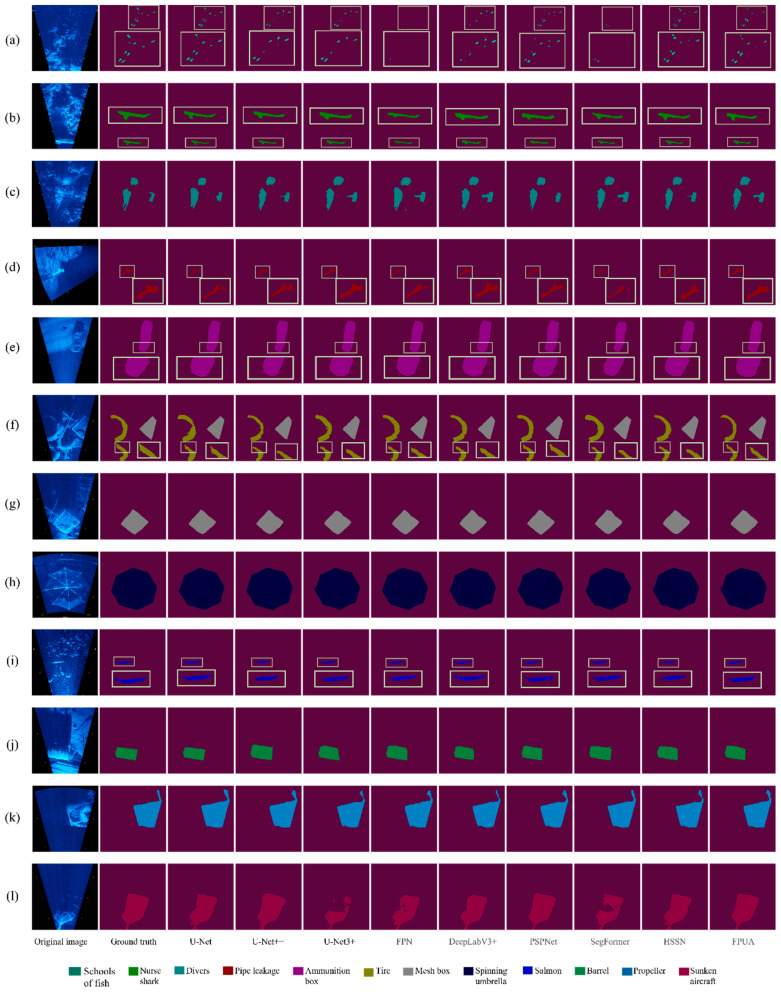
Segmented images in real oceans. Subfigure (**a**) shows the segmentation effect of a school of fish, subfigure (**b**) shows the segmentation effect of a nurse shark, subfigure (**c**) shows the segmentation effect of divers, subfigure (**d**) shows the segmentation effect of pipe leakage, subfigure (**e**) shows the segmentation effect of an ammunition box, subfigure (**f**) shows the segmentation effect of tires, subfigure (**g**) shows the segmentation effect of a mesh box, subfigure (**h**) shows the segmentation effect of spinning umbrella, subfigure (**i**) shows the segmentation effect of salmon, subfigure (**j**) shows the segmentation effect of barrel, subfigure (**k**) shows the segmentation effect of propeller, and subfigure (**l**) shows the segmentation effect of sunken aircraft. The yellow box in the figure represents the enlargement of the details.

We divided the dataset according to 8:1:1. The data were randomized for each experiment and the experiments have been repeated 10 times to obtain the average experimental results, which fully meet the requirements of cross-validation. We compared our model with other models and obtained the results, as shown in Figure 14. Regarding the small object classes (Figure 14a,b,d,h), the contours of our model are closer to the original image. Moreover, on the other classes, the segmentation is more natural.

By analyzing Table 7, in the small object class, the FPUA model improves in two classes, schools of fish, and pipe leakage, but is 1% worse than the best model in the salmon class in terms of accuracy. In the mIoU of these four small object classes, our model can outperform the other models by at least 1.9% in segmentation accuracy. In the other classes, the FPUA model is 0.6% worse than the best model in the drivers’ class, 0.5% behind the best model in the tires class, and 0.8% worse than the best model in the spinning umbrella class. In the propeller class, it is about 0.6% worse than the best model; however, FPUA achieves the best segmentation accuracy in the four classes of ammunition box, mesh box, barrel, and sunken aircraft. The FPUA model also achieves 80.52% accuracy in the mIoU index, which is 1.3% ahead of other effective models, which proves that FPUA model can achieve good segmentation accuracy in real underwater environment.

### 4.6. Real Forward-Looking Sonar System Dataset

**Figure 15:**
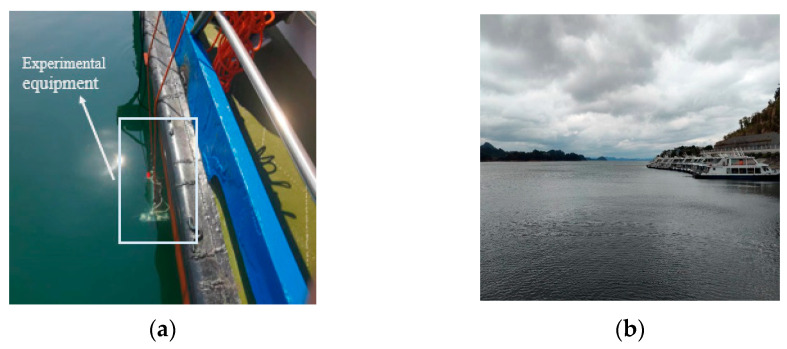
Qiandao Lake experimental environment. (**a**) Placement of experimental vessel and equipment. (**b**) There are several boats docked at the lake, and there are steps on the side of the boats.

**Figure 16:**
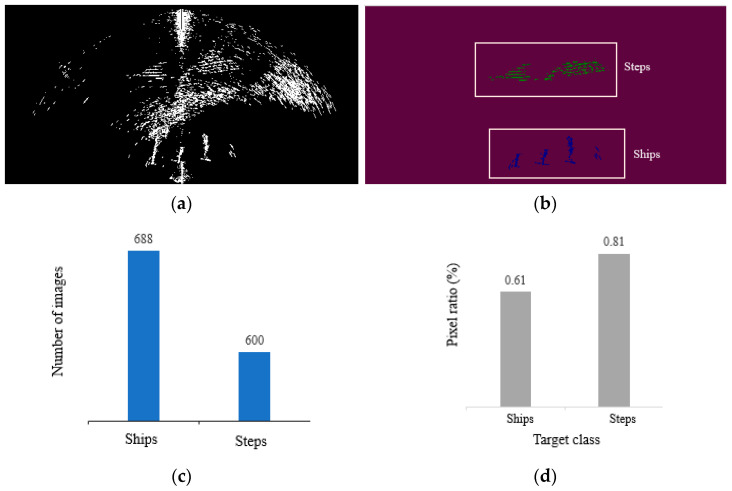
Data for each class in the image. (**a**) Acquired sonar images. (**b**) The green part of the marked image information is the step, and the blue part is the ship. (**c**) Number of images accounted for by each class. (**d**) The average of the ratio of pixels occupied by that class in the image where that class exists.

To verify the performance of the model under the actual forward-looking sonar equipment, we conducted experiments in Qiandao Lake using our self-developed forward-looking sonar equipment. The device operates at 350 KHz, with a detection distance of 25 m and an opening angle of 135° and a total of 512 beams. The equipment is installed on the side of the test vessel to collect sonar data from the outside, as shown in Figure 15. As the ship moves, we acquire an image every three seconds. A dataset containing 1000 images was produced with data from both the step and ship classes. The number of images and pixel distribution for each class are shown in Figure 16. Compared with the other two datasets, our collected data show a longer distance, so the pixel share of objects is smaller, and it can also be found from Figure 16a that our data have more serious noise interference, which better reflects the sonar images in complex scenes.

By analyzing the images as well as the data, we can find that the steps and ships occupy a small percentage of pixels and belong to the small object class. At the same time, there is a great deal of noise in our acquired images, so we also need to consider the recognition of our model regarding noisy images.

### 4.7. Real Forward-Looking Sonar System Experimental Results

By analyzing the data in Table 8, it is evident that FPUA can achieve better results when dealing with both ships and steps. Our model can improve the segmentation progress by 1.6% on the ship class and can improve the segmentation accuracy by at least 1.3% on the step class compared to other models. This further demonstrates that our model has good segmentation results in forward-looking sonar images.

## 5. Conclusions

This study proposed a semantic segmentation network model FPUA for forward-looking sonar images. The model uses U-Net as the backbone and combines a residual block to increase the depth of the network that can be trained effectively. Then, the FPN module combined with attention was introduced, which improves the segmentation accuracy of the network model for small-object classes and also has a good segmentation effect for few-sample classes. In the water tank environment, FPUA had a great advantage in forward-looking sonar image segmentation and achieved better segmentation for few-sample and small-object classes. Specifically, the proposed model improved the average segmentation accuracy by 1.5% for the few-sample classes and 1% for the small-object classes. The proposed model achieved a segmentation accuracy of 73.8%, which is 1.3% higher than other semantic segmentation models. In the real environment data, FPUA also outperformed other models by at least 1.3% in average segmentation accuracy, which achieved a segmentation result of 80.52%. In the data collected by our self-developed device, despite the presence of relatively severe noise interference, the segmentation accuracy of FPUA can also be improved by 1.26% to 65.66% compared to other effective models.

FPUA focuses on the problem of object feature extraction under noise interference, and the three datasets also represent different environments and different noise interference. Compared with other models, our model achieves better results on all three datasets. In addition, experiments on multiple datasets show that the model can be applied to sonar images under different noises. Further, the model can also achieve better results on other sonar devices, such as high-resolution synthetic aperture sonar, by virtue of its feature extraction capability.

In future research, we will further investigate object boundary segmentation and realize a more refined semantic segmentation model for forward-looking sonar images. We will also conduct experiments on different types of sonar devices to further confirm the segmentation accuracy of the model.

## Figures and Tables

**Figure 11:**
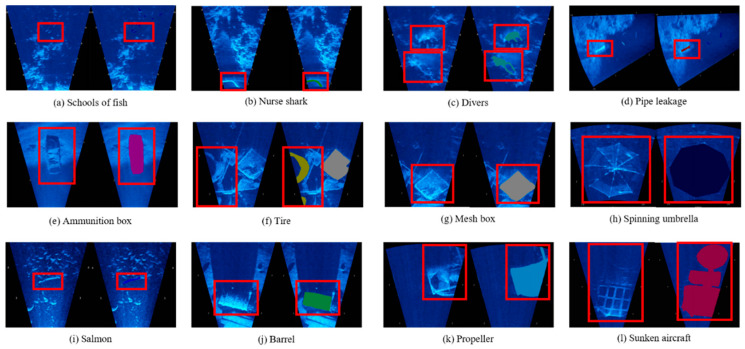
Images of each class and their labeled information images. The red box in each subfigure indicates the location of the objects in that class, and different colors are used to show the different classes, as shown in subfigures (**a**–**l**).

**Table 1:** Dataset statistics.

Dataset	Quality	Resolution	Train-Test Split
Tank dataset	1868	320 × 480	1000 for train, 617 for test, 251 for verification
Marine dataset	3116	320 × 320	2493 for train, 312 for test, 311 for verification
Self-developed equipment datasets	1000	512 × 256	800 for train, 100 for test, 100 for verification

**Table 2:** Comparison of different architectures implementation.

Model	Parameters
U-Net [39]	14.3 M
U-Net++ [50]	31.4 M
U-Net3+ [51]	26.7 M
FPN [49]	46.1 M
DeepLabV3+ [54]	22.9 M
PSPNet [52]	2.8 M
Segformer [57]	47.3 M
HSSN [58]	88.5 M
FPUA	14.4 M

**Table 3:** IoU for each class in the test set for investigation of segmentation model performance. The best segmentation accuracy for each class is marked bold.

Model	U-Net [39]	U-Net++ [50]	U-Net3+ [51]	FPN [49]	DeepLabV3+ [54]	PSPNet [52]	SegFormer [56]	HSSN [57]	FPUA
Bottle	0.631	0.728	0.723	0.731	0.683	0.746	0.643	**0.749**	0.741
Can	0.593	0.563	0.516	0.562	0.607	**0.622**	0.522	0.582	0.620
Chain	0.622	0.632	0.605	0.618	0.568	0.535	0.575	0.633	**0.641**
Drink carton	0.738	0.711	**0.744**	0.693	0.658	0.691	0.644	0.735	0.742
Hook	0.693	0.710	0.695	0.717	0.630	0.638	0.595	**0.747**	0.731
Propeller	0.650	0.686	**0.706**	0.694	0.694	0.692	0.633	0.699	0.705
Shampoo bottle	0.816	0.767	0.822	0.590	0.818	0.821	0.674	0.832	**0.844**
Standing bottle	0.640	**0.779**	0.677	0.550	0.748	0.693	0.582	0.723	0.778
Tire	0.869	0.880	0.875	0.858	0.875	0.870	0.833	0.859	**0.888**
Valve	0.378	0.509	0.501	0.538	0.521	0.510	0.361	**0.566**	0.557
Wall	0.859	**0.876**	0.869	0.872	0.862	0.865	0.859	0.847	0.868
Few-sample mIoU	0.700	0.736	0.725	0.638	0.722	0.711	0.621	0.750	**0.765**
Small-object mIoU	0.653	0.695	0.688	0.618	0.675	0.671	0.571	0.721	**0.730**
mIoU	0.680	0.713	0.703	0.675	0.697	0.699	0.629	0.725	**0.738**

**Table 4:** Modules included in different models.

Modules	U-Net	Model 1	Model 2	Model 3	Model 4	FPUA
Residual block		√	√			√
Feature Pyramid module			√	√	√	√
Attention Structure					√	√

**Table 5:** Segmentation results for ablation experiment. The best segmentation accuracy for each class is marked bold.

Model	U-Net	Model 1	Model 2	Model 3	Model 4	FPUA
Bottle	0.631	0.731	**0.746**	0.727	0.738	0.741
Can	0.593	0.565	0.607	0.609	0.576	**0.620**
Chain	0.622	**0.641**	0.584	0.593	0.606	**0.641**
Drink carton	0.738	0.738	0.740	0.715	0.729	**0.742**
Hook	0.693	0.707	0.709	0.675	0.685	**0.731**
Propeller	0.650	0.665	**0.707**	0.676	0.678	0.705
Shampoo bottle	0.816	0.811	0.829	0.793	0.817	**0.844**
Standing bottle	0.640	0.776	0.777	0.762	**0.778**	**0.778**
Tire	0.869	0.879	0.858	0.833	0.887	**0.888**
Valve	0.378	0.510	0.544	0.523	0.543	**0.557**
Wall	0.859	0.866	0.866	0.867	**0.870**	0.868
mIoU	0.680	0.717	0.724	0.707	0.719	**0.738**

**Table 7:** The mIoU results for each class of each model under real data. The best segmentation accuracy for each class is marked bold.

Model	U-Net [39]	U-Net++ [50]	U-Net3+ [51]	FPN [49]	DeepLabV3+ [54]	PSPNet [52]	SegFormer [56]	HSSN [57]	FPUA
Schools of fish	0.551	0.572	0.587	0.572	0.625	0.581	0.497	0.611	**0.635**
Nurse shark	0.679	0.698	0.682	0.659	0.662	0.696	0.622	**0.721**	0.716
Divers	0.633	0.643	0.639	0.617	**0.691**	0.671	0.598	0.679	0.685
Pipe leakage	0.689	0.694	0.704	0.522	0.695	0.747	0.611	0.733	**0.754**
Ammunition box	0.884	0.898	0.872	0.890	0.894	0.909	0.883	0.903	**0.939**
Tire	0.613	0.623	**0.633**	0.577	0.588	0.587	0.592	0.618	0.628
Mesh box	0.946	0.958	0.950	0.932	0.924	**0.963**	0.944	0.946	0.958
Spinning umbrella	**0.979**	0.970	0.971	0.958	0.960	0.961	0.913	0.954	0.971
Salmon	0.659	**0.715**	0.684	0.597	0.679	0.643	0.657	0.713	0.704
Barrel	0.852	0.863	0.858	0.863	0.851	0.866	0.833	0.852	**0.873**
Propeller	0.943	0.947	0.935	0.921	**0.960**	0.940	0.917	0.948	0.954
Sunken aircraft	0.809	0.821	0.814	0.812	0.832	0.819	0.822	0.833	**0.844**
Small object mIoU	0.645	0.670	0.664	0.588	0.665	0.667	0.597	0.655	**0.674**
mIoU	0.770	0.783	0.777	0.743	0.780	0.782	0.741	0.792	**0.805**

**Table 8:** IoU of each class in the images acquired by the self-developed equipment. The best segmentation accuracy for each class is marked bold.

Model	U-Net [39]	U-Net++ [50]	U-Net3+ [51]	FPN [49]	DeepLabV3+ [54]	PSPNet [52]	SegFormer [56]	HSSN [57]	FPUA
Ships	0.5204	0.5801	0.6017	0.5192	0.5907	0.5455	0.4977	0.6022	**0.6183**
Steps	0.6061	0.6461	0.6325	0.5569	0.6491	0.6693	0.5338	0.6837	**0.6952**
mIoU	0.5634	0.6131	0.6171	0.5381	0.6199	0.6074	0.5158	0.6430	**0.6566**

## Data Availability

The water tank data are presented by Alejandro et al. and the dataset is available at https://github.com/mvaldenegro/marine-debris-fls-datasets/, which was accessed on 1 August 2022. The marine dataset is from the website http://www.soundmetrics.com/, which was accessed on 15 March 2022. We used the website data to make our own dataset. The real forward-looking sonar system dataset is not ready to publish.
